# Inflammasome in Platelets: Allying Coagulation and Inflammation in Infectious and Sterile Diseases?

**DOI:** 10.1155/2015/435783

**Published:** 2015-02-26

**Authors:** Eugenio D. Hottz, Ana Paula T. Monteiro, Fernando A. Bozza, Patrícia T. Bozza

**Affiliations:** ^1^Laboratório de Imunofarmacologia, Instituto Oswaldo Cruz, Fundação Oswaldo Cruz, 21040-900 Rio de Janeiro, RJ, Brazil; ^2^Laboratório de Medicina Intensiva, Instituto Nacional de Infectologia Evandro Chagas, Fundação Oswaldo Cruz, 21040-900 Rio de Janeiro, RJ, Brazil; ^3^Instituto D'Or de Pesquisa e Ensino, 22281-100 Rio de Janeiro, RJ, Brazil

## Abstract

Platelets are crucial effector cells in hemostasis. In addition, platelets are increasingly recognized as major inflammatory cells with key roles in innate and adaptive immune responses. Activated platelets have key thromboinflammatory activities linking coagulation to inflammatory response in a variety of coagulation disorders and vasculopathies. Recently identified inflammatory activities of platelets include the synthesis of IL-1*β* from spliced pre-RNA, as well as the presence and assembly of inflammasome which intermediate IL-1*β* secretion. Here we review the mechanisms by which platelets activate translation machinery and inflammasome assembly to synthesize and release IL-1*β*. The contributions of these processes to protective and pathogenic responses during infectious and inflammatory diseases are discussed.

## 1. Introduction

Platelets are classically known as essential and specialized effector cells in hemostasis. In large part due to their anucleate status, the activities attributed to platelets were for a long time restricted to their procoagulant and wound healing functions, with rapid responses involving aggregation and granule secretion. Nevertheless, recent studies have shown a new repertoire of platelet activities mainly related to immune and inflammatory responses [1, 2]. Even though platelets do not have nucleus, they have stored RNA molecules and diverse mechanisms for posttranscriptional process RNA using specialized pathways to change their proteome, phenotype, and functions [3]. In addition, platelets are relatively long-living cells and can mediate responses for many hours after initial adhesion, aggregation, and secretion. Consonant with the recent advance in platelet biology, newly recognized inflammatory activities continue to emerge in platelets. An important find in this field was the recent demonstration of platelets assembling of functional inflammasome to process and secrete biologically active interleukin- (IL-) 1*β* [4].

IL-1*β* is the best described newly synthesized protein after pro-RNA splicing in platelets [3, 5, 6]. This cytokine is linked to endothelial dysfunction and coagulation disorders in many inflammatory, infectious, and cardiovascular diseases [7–10]. It activates cells of the immune system and of the vascular wall through IL-1 receptor (IL-1R) signaling promoting inflammation, angiogenesis, and differentiation of myeloid progenitor cells [11]. IL-1*β* activity in activated platelets has been extensively reported in the last two decades [5, 6, 12–15], including the pathways involved in IL-1*β* synthesis [3, 5, 6]. However, the pathways involved in processing and secretion of IL-1*β* by platelets, including requirement of inflammasome, were only recently dissected [4, 6, 16]. Here we provide an overview of platelet inflammatory responses that require IL-1*β* signaling and inflammasome activation. We emphasize the potential implication of these pathways to inflammatory activities of platelets in diverse vasculopathies and coagulopathies in response to pathogens or sterile stimuli.

## 2. Inflammasome Signaling in Platelets

**Figure 1:**
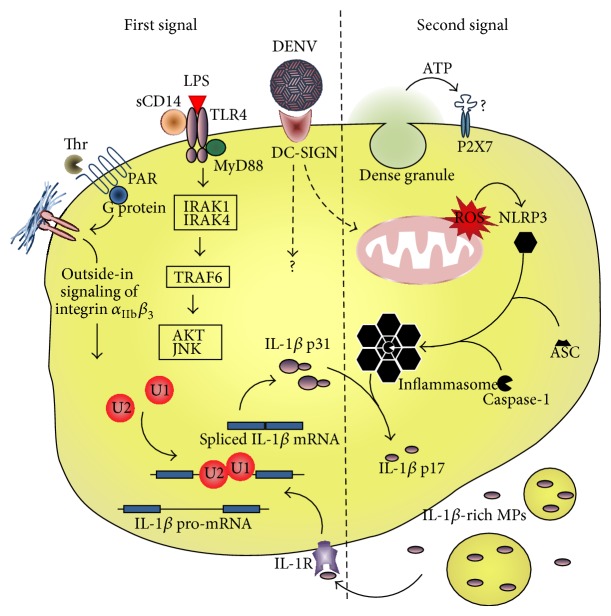
Inflammasome signaling in platelets. Schematic representation of the pathways leading to IL-1*β* synthesis (first signaling, left) and inflammasome assembly (second signaling, right) in platelets in response to procoagulant or pathogen-associated stimuli.

Inflammasome activation in nucleated cells usually needs two distinct signal cascades from pattern recognition receptors (PRR). The first one, also termed priming, culminates in translocation of NF*κ*B to the nucleus and synthesis of IL-1*β*, IL-18, and the inflammasome components. The second signal follows the activation of a cytosolic recognition system of pathogen-associated or damage-associated molecular patterns (PAMP and DAMP, resp.). The better characterized inflammasome uses NOD-like receptor containing domain pyrin 3 (NLRP-3) as sensor, which recruits the adaptor apoptosis-associated speck-like protein containing a CARD domain (ASC) and the proteolytic subunit caspase-1 to assemble the inflammasome and process IL-1*β* and/or IL-18 into mature cytokines [17–19]. We recently described that platelets constitutively express the inflammasome components NLRP3 and ASC and can use them to assemble functional inflammasome, activate caspase-1, and process IL-1*β* [4]. In addition to constitutively expressed proteins, inflammasome components are also detected at the transcriptional level in platelets [20]. The signaling pathways for IL-1*β* synthesis and inflammasome activation demonstrated in platelets up to the moment are highlighted here and summarized in Figure 1.

As aforementioned, platelets have stored RNA molecules; many of them present as pre-RNA and need spliceosome-dependent mechanisms for their processing into mature transcripts [3, 21]. Signal-dependent splicing of IL-1*β* pre-mRNA in activated platelets is controlled by cdc-like Kinase 1 (CLK1) [6, 22]. This mechanism is activated by thrombin or other classic agonists in the presence of fibrinogen and requires outside-in signaling from integrin *α*_IIb_ *β*_3_ engagement to fibrin during clot formation [3, 5, 21]. CLK-1-mediated IL-1*β* RNA splicing also occurs after Toll-like receptor- (TLR-) 2- and TLR-4-mediated platelet activation by Pam3Cys or LPS, respectively [6, 16, 22]. As in nucleated cells, LPS engagement to TLR-4 on platelets leads to recruitment of MyD88 adaptor and phosphorylation of IRAK1 and 4 [6]. TRAF6 couples MyD88 signaling to AKT/JNK pathway, leading to IL-1*β* RNA splicing in LPS-stimulated platelets [6]. As platelets themselves do not express CD14 [23], LPS-induced IL-1*β* RNA processing is potentiated in the presence of sCD14 [22]. Surprisingly, anucleate platelets have transcription factors as NF*κ*B and STAT3, which present nontranscriptional activities in these cells [24, 25]. LPS or Pam3Cys activation of TLR-4 or -2 on platelets results in I*κ*B degradation and NF*κ*B-dependent platelet responses [24]. However, the requirement of NF*κ*B for IL-1*β* synthesis in platelets remains to be determined.

Platelets express the lectin receptor dendritic cell-specific ICAM-3-grabbing nonintegrin (DC-SIGN) which recognizes pathogen-associated carbohydrates [26, 27]. DC-SIGN also recognizes glycosylated domains on the envelope protein of dengue virus (DENV) [28, 29], and DENV activates platelets depending on DC-SIGN expression [30]. We have recently demonstrated that platelet activation by DENV induces IL-1*β* synthesis and secretion [4]. In this study [4] we showed through flow cytometry and confocal fluorescence microscopy that freshly isolated platelets constitutively express the inflammasome components NLRP3 and ASC, which were dissociated in rested platelets but colocalized in activated platelets from patients with dengue, indicating presence of assembled inflammasomes during dengue disease. This was consistent with increased caspase-1 activity in platelets from patients or platelets stimulated with DENV* in vitro* and the ability of YVAD-fmk caspase-1 inhibitor to impair IL-1*β* secretion by DENV-activated platelets. Finally, cleaved IL-1*β* was detected in platelets from DENV-infected patients using western blot analysis [4]. These observations of platelets from patients with dengue, as well as from* in vitro* infection models and functional assays, provided the first evidence for inflammasome activity in platelets, and its assembly during DENV infection culminating in the release of IL-1*β*.

Inflammasome activation in DENV-stimulated platelets required as second signaling the generation of reactive oxygen species (ROS) in mitochondria [4], consistent with ROS-mediated NLRP3 inflammasome activation in monocytes [19, 31]. Curiously, caspase-1-dependent IL-1*β* processing in thrombin- or LPS-stimulated platelets do not require the addition of a second stimulus [5, 6, 16, 22] as required by LPS-primed nucleated cells [17–19]. Platelet activation induces secretion of ATP from dense granules [24, 32, 33] and NLRP3-inflammasome responds to extracellular ATP [34]. Mitochondria-derived ROS and/or ATP from dense granule are likely involved in inflammasome activation after procoagulant or TLR stimulation.

Platelet-produced IL-1*β* is chiefly secreted in microparticles (MP), as after thrombin stimulation [5], as in PRR activation by LPS or DENV [4, 6, 16]. Interestingly, shedding of IL-1*β*-containing MPs is damped by caspase-1 inhibitors [4, 16], demonstrating that IL-1*β* packaging and secretion in MPs are uncoupled from IL-1*β* synthesis and depend on active inflammasomes. This notion was reinforced by Brown and McIntire [6], who showed that TRAF6 activation induces AKT/JNK phosphorylation and IL-1*β* synthesis without inducing the shedding of MPs.

Platelets and megakaryocytes express IL-1R on surface and respond to IL-1*β* [16, 35]. IL-1*β* stimulates megakaryocyte maturation and enhances platelet aggregation and adhesion in response to collagen and fibrinogen [35]. The signaling cascade after IL-1*β*-IL-1R engagement is very similar to TLR agonist stimulation [6, 24, 35]. Thus, IL-1*β* signals its own synthesis in platelets and an IL-1*β* autocrine loop potentiate IL-1*β* synthesis and shedding of IL-1*β*-rich MPs in LPS-activated platelets [16].

## 3. Platelet Inflammasome in Sterile Thrombosis and Inflammation

Platelets become activated by contact with matrix proteins and/or von Willebrand factor exposed on endothelial cells after interaction with injured endothelium [36–38]. This initial procoagulant response is amplified by platelet-secreted agonists and by adhesion of integrin *α*_IIb_ *β*_3_ to fibrin mesh [37, 39]. During this process, platelets synthesize, process, and secrete IL-1*β* in response to different procoagulant stimuli including endothelial matrix proteins (collagen) and secreted agonists (thrombin, ADP, epinephrine and PAF) [5]. Part of the IL-1*β* synthesized in platelets remains as cell-associated cytokine [5]. Activated platelets interact with and signal inflammatory responses to endothelial cells; many of these responses involve IL-1*β* signaling [13, 14, 38]. Importantly,* in vitro* models for clot formation and retraction show IL-1*β* accumulation in platelet-fibrin clump [5] and IL-1*β* is found in occlusive thrombus* in vivo* few hours after damage of vascular wall induced by FeCl_3_ [16]. IL-1*β* accumulation in arterial thrombosis* in vivo* precedes mononuclear leukocytes incorporation to the clot and is not affected by transcription inhibition, which dampens IL-1*β* synthesis in leukocytes but not in platelets [16]. Thus, platelet-generated IL-1*β* accumulates in sterile thrombi allying coagulation to local inflammation of the endothelium. However, whether and how inflammasomes are activated in platelets under sterile thrombosis deserve further investigation.

When endothelial cells are exposed to activated platelets or conditioned medium from activated platelets they release cytokines and chemokines including IL-6, IL-8, MCP-1, and GM-CSF [13, 14, 40]. This signaling is blocked by IL-1R antagonist [13, 14]. Moreover, during vascular injury platelets and platelet products gain access to the extravascular milieu, and vascular smooth muscle cells secrete proinflammatory cytokines in response to IL-1*β* from platelets [12]. Endothelial cells exposed to IL-1*β*-expressing platelets or platelet-derived MPs express the adhesion molecules ICAM-1 and VCAM-1 depending on IL-1R [6, 14] and support the adhesion of polymorphonuclear (PMN) neutrophils [5]. IL-1R-mediated neutrophils transendothelial migration takes place after activation of brain microvascular EC monolayers with supernatant from activated platelets, and neutrophils infiltration to brain tissue occurs after cerebral ischemia* in vivo* close to ICAM-1-expressing vessels with adherent platelets [41].

Infiltration of neutrophils and platelet-derived MPs are also observed in the synovial fluid from patients with rheumatoid arthritis [42]. Analyses of experimental rheumatoid arthritis together with* in vitro* models for platelet-synoviocyte interaction indicate that platelets become activated in the inflamed joint by local exposure to collagen or collagen-producing fibroblast-like synoviocytes (FLS). These platelets shed IL-1*α*- and IL-1*β*-containing MPs which reciprocally activate resident fibroblast-like synoviocytes in the synovial space. MPs-activated synoviocytes secrete proinflammatory cytokines and chemokines including IL-6, IL-8, and MCP-1, amplifying inflammation in synovial compartment [42].

## 4. Platelet Inflammasome in Infectious Diseases

Infectious disease continues to be a leading cause of death globally. Platelet activation, including shedding of MPs, plays pathogenic roles in several clinical conditions such as dengue, sepsis, malaria, and HIV/AIDS syndromes [4, 30, 43–46]. Among many ways in which platelets can intermediate inflammatory and immune responses in infectious diseases [1, 2] recent evidence highlights the roles for inflammasome in platelets [4] and the relevance of platelets as main sources of IL-1*β* [47]. Contributions of platelet IL-1*β* synthesis and inflammasome activation to infectious diseases models are summarized here.

It has been demonstrated elsewhere [22] that LPS (even at low concentrations) can start IL-1*β* RNA processing in platelets, with translation and accumulation of IL-1*β* protein. Comparing the synthesis and accumulation of IL-1*β*, LPS exhibits a more robust response than classical platelet agonists as thrombin and collagen [5]. Because gram negative bacterial sepsis is a major clinical problem with few therapeutic options, much interest has been on the implications for LPS activation of platelet TLR4 to the pathogenesis of sepsis and endotoxemia [6, 16, 48]. Shedding of IL-1*β*-containing MPs is a major response to LPS in platelets [6, 16]. IL-1*β*-containing MPs recovered from LPS-stimulated platelets activate IL-1R on endothelial cells leading to VCAM-1 expression [6]. Endothelial activation with compromising of cardiovascular system is a leading cause of shock during severe sepsis. Inflammation participates in this process and is associated with local thrombosis. Considering the endothelial role in cardiovascular aspect of sepsis, MPs-induced activation of endothelium uncovers a potential role for platelet IL-1*β* in septic syndrome.

During experimental cerebral malaria in mice, activated platelets are the main source of circulating IL-1*β* [47]. Malaria is a tropical infectious disease caused by mosquitos-spread* Plasmodium* parasites. Inflammatory vasculopathy is a feature of severe malaria, which includes cerebral malaria and pulmonary malaria. Sequestration of platelets with leukocytes and parasitized red blood cells in the vascular bed with activation of the coagulation cascade and disruption of endothelial barrier function are thought to contribute to vasculopathy in severe malaria [49, 50]. Platelet activation and thrombocytopenia occur early in complicated malaria [47, 51]. In murine model for cerebral malaria, early platelet activation is protective by inducing the acute phase response and limiting parasite burden [47], while continued platelet activation is detrimental to the host probably by contributing to vasculopathy [52]. Platelet-induction of acute phase response at the liver depends on IL-1*β* synthesis and secretion by platelets [47], highlighting the potential role for platelet inflammasome in protective responses during severe malaria.

Dengue is an arthropod-born viral disease caused by the four dengue virus serotypes (DENV1–4). DENV infection induces a spectrum of clinical manifestations that range from mild self-limited dengue fever to severe dengue, a life-threatening syndrome associated with increased vascular permeability, hypovolemia, hypotension, and shock [53, 54]. Thrombocytopenia is commonly observed in dengue syndromes and correlates with the onset of plasma leakage and with risk for severe dengue [7, 54–57]. IL-1*β* is an important proinflammatory cytokine increased during severe dengue [7, 8, 58]. Of note, increased IL-1*β* levels associate with thrombocytopenia, increased endothelial permeability [7], thrombosis, and dysregulated hemostasis in dengue disease [8]. We recently reported increased expression of IL-1*β* in platelets from patients with dengue and in platelets exposed to DENV* in vitro*. We demonstrated that dengue induces NLRP3-inflammasome assembly and caspase-1-dependent IL-1*β* secretion in platelets. IL-1*β* was also detected in platelet MPs from patients with dengue and in MPs from platelets activated by DENV* in vitro*. Importantly, platelet synthesis of IL-1*β*, inflammasome activation, and shedding of IL-1*β*-containing MPs were correlated with clinical signs of increased vascular permeability in dengue patients, and MPs recovered from DENV-activated platelets increased endothelial cell permeability* in vitro* depending on IL-1R [4]. This translational study demonstrates that DENV-triggered IL-1*β* synthesis in platelets is a mechanism for endothelial activation and increased vascular permeability in dengue syndrome.

## 5. Conclusion

**Figure 2:**
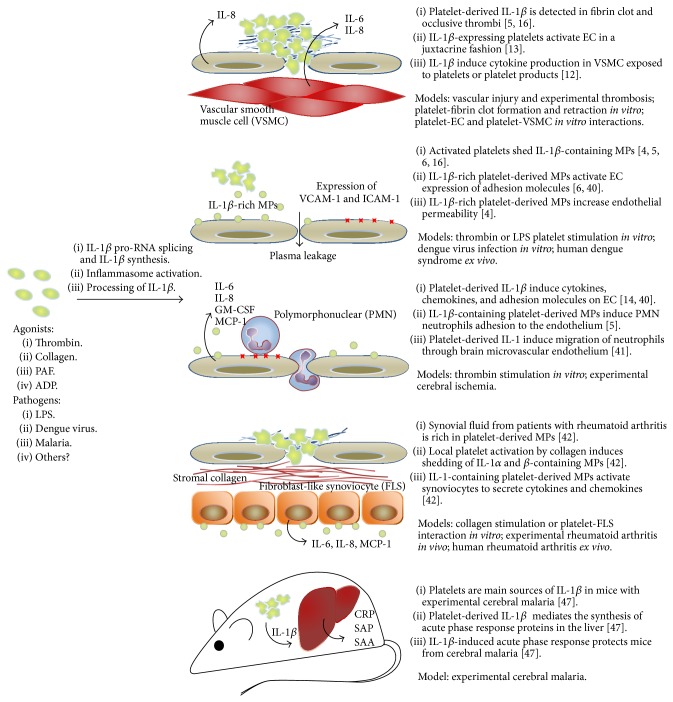
Overview of the consequences for platelet-derived IL-1*β* and inflammasome activation in thromboembolic, infectious, and inflammatory diseases. EC: endothelial cell; MPs: microparticles; CRP: C reactive protein; SAP: serum amiloid P; SAA: serum amiloid A.

Inflammasome components and mechanisms for inflammasome activation were only recently described in platelets, which opens new perspectives and research opportunities in diseases pathogenesis. As discussed above, IL-1*β* synthesis and inflammasome processing of IL-1*β* in platelets are implicated in a range of inflammatory and infectious conditions. The immune and inflammatory activities of platelets that require inflammasome activation and IL-1*β* signaling are summarized in Figure 2. This review highlighted our basic knowledge on platelet inflammasome and the signaling cascades needed to its activation. Currently, there is little information regarding inflammasome in platelets. While NLRP3 inflammasome was recently demonstrated in platelets [4], a range of inflammasomes that respond to different inflammatory stimulus and pathogens remain to be described in these important effector cells. Synthesis of IL-1*β* by platelets and IL-1*β* shedding in platelet MPs have been implicated in diverse pathologies including inflammatory, thromboembolic, and infectious diseases [4, 16, 42, 47]. However, conditions in which platelet inflammasomes play pathogenic and/or protective roles still deserve more in-depth investigation. Commitment to this investigative front will undoubtedly identify new hemostatic and inflammatory roles for platelet IL-1*β* and inflammasome.
